# Norm compliance affects perceptual decisions through modulation of a starting point bias

**DOI:** 10.1098/rsos.171268

**Published:** 2018-03-28

**Authors:** Ulf Toelch, Folco Panizza, Hauke R. Heekeren

**Affiliations:** 1 Biological Psychology and Cognitive Neuroscience, Freie Universität Berlin, Habelschwerdter Allee 45, 14195 Berlin, Germany; 2 QUEST Center, Berlin Institute of Health, Anna-Louisa-Karsch-Strasse 2, 10178 Berlin, Germany; 3 Center for Mind/Brain Sciences (CIMeC), University of Trento, Via delle Regole 101, 38123 Mattarello, Italy

**Keywords:** conformity, drift-diffusion models, social information, norms, social influence, decision-making

## Abstract

Adaptive decisions in social contexts depend on both perceptual information and social expectations or norms. These are potentially in conflict when certain choices are beneficial for an individual, but societal rules mandate a different course of action. To resolve such a conflict, the reliability of information has to be balanced against potentially deleterious effects of non-compliance such as ostracism. In this study, we systematically investigated how interactions between perceptual and social influences affect decision-relevant cognitive processes. In a direction-of-motion discrimination task, participants received perceptual information alongside information on other players' choices. In addition, we created conflict scenarios where players’ choices affected other participants' monetary rewards dependent on whether their choices were in line or against the opinion of the other players. Importantly, we altered the strength of this manipulation in two separate experiments by contrasting motivations of either preventing harm or providing a benefit to others. Behavioural analyses and computational models of perceptual decisions showed that participants successfully integrated perceptual with social information. Participants' reliance on social information was effectively modulated in conflict situations. Critically, these effects were augmented when the strength of social norms was increased, indexing conditions under which social norms effectively influence decisions. These results inform theories of social influence by providing an account of how higher order goals like social norm compliance affect perceptual decisions.

## 1. Introduction

Adaptive decisions often require integration of information from several perceptual sources that are weighted according to their respective reliability [1,2]. This perceptual information can be effectively combined with information acquired from social sources (henceforth social information) via observation of or interaction with others [3–6]. For example, when crossing a street one can combine information from accurate visual information (not seeing a car in the looking direction), less accurate auditory information (but hearing a car), and observing other pedestrians abruptly stopping before crossing the street, looking into the opposite direction. The combined information will then delay one's own crossing until the car has passed. The interplay between potentially conflicting perceptual and social information creates population-level phenomena like herding [7,8] or conformity [9] that affect cultural evolutionary processes [10,11].

Beyond choice relevant information, social norms guide decisions. That is, societal rules or expectations exert a strong influence on behaviour. Non-compliance with these rules is sanctioned [12–14] or results in a reduction of social reputation and potential ostracism [15,16]. How strongly norms affect decisions is thus influenced by how likely and severe sanctions are [17–20].

Consequently, both an optimality goal and a compliance goal underpin decisions in social contexts. Whereas the former aims at adjusting behaviour given the available information, the latter is concerned with the stability of social relationships [21]. The possible interaction between the two goals has long been recognized [22], but social norms remain understudied [23] and a systematic investigation that varies available information (perceptual and social) alongside social norms has not been conducted [24]. This is particularly important as many policy interventions aim at changing social norms and beliefs [25]. These interventions often address severe societal problems to reduce for example health-damaging behaviour like excessive drinking [26] or violence [27]. Here, a deeper understanding of the psychological processes will facilitate developing interventions for behavioural change [18,28].

In the present research, we ask how social norms and choice relevant information are balanced in decisions. As outlined above, this balance will depend on the interaction between three factors: uncertainty of available perceptual and social information, the strength of the social norm, and the conflict between the two. In line with previous research, we predict that participants will integrate perceptual and social information to inform their decisions and reduce uncertainty [5,29–32]. At the same time, social norms have an increased impact on decisions under high information uncertainty [33,34]. Hence, decisions in line with social information are potentially elicited by available perceptual and social information, social norms or both [24]. A segregation of the two effects is important for identifying the unique contribution of social norms on decisions.

We investigated the interaction between information (perceptual and social) and social norms in an established two alternative forced choice (AFC) perceptual task (direction-of-motion discrimination) where players received monetary rewards for correct answers. Additionally to the perceptual stimulus (motion energy, i.e. *perceptual information*), we provided players with decision information from two players that simultaneously played the same random dot task in the same room (*social information*). Conflict or concordance between the two information types created conditions with low and high information reliability through the integration of the two information types. We were thus able to judge how participants decided based on perceptual and social information only. Our main question was how this balance was influenced by social norms. We implemented *social norms* via rules that prompted participants to decide with or against the presented social information in particular trials. To ensure that norm compliance had consequences on a social level, norm compliance affected the remuneration of the *other* players (see below). That is, under uncertain conditions, participants could be swayed to decide in line with the norm. Critically, this manipulation uncoupled norm compliance from following social information due to an optimality goal, i.e. using social information to reduce perceptual uncertainty. Hence, we predict that social norms affect players' accuracy over and beyond the validity of the social information. Moreover, our norms acted in two directions, either to decide with or against the social information. This allowed us to investigate how social norms influence decisions if they are in line with the social information (e.g. in conformity) and if social norms also influence choices if they are not aligned with social information. We predict that choices in line with social information are affected by social norms bi-directionally depending on concordance or conflict with the presented social information.

To disentangle which specific components of the perceptual decision-making process were actually under the normative or informational influence, we applied established drift-diffusion models (DDMs) to choice and reaction time data that allow for an in-depth investigation of perceptual decision-making processes [35–38]. Previous work suggests that prior information about the reliability or profitability of a response biases participants’ choices by shifting the starting point in the direction of the preferred option [39–41]. That is, less evidence is needed to elicit a response in the direction of the preferred option. There is also some evidence that drift rates, amount of evidence accumulated per time unit, instead of starting points are potentially affected in social contexts [42,43]. Here, we explicitly tested to what extent social information elicited a starting point bias or a change in drift rates. These two factors will change the amount of evidence (perceptual information) that is needed to elicit a response. In a second step, we were interested in how such changes through social information were then modulated by social norms.

As an additional test for the influence of norms, we varied the strength of social norms through a manipulation of the outcome of norm (non)-compliance across two experimental groups. In the *harm to others* experiment, non-compliance to norms resulted in a deduction of money from the other players. In the *benefit to others* experiment, compliance resulted in additional money for the other players. This was motivated by the general notion that humans are more sensitive to losses than to gains [44]. In social contexts, causing a loss for a third party is considered more harmful and less prosocial than to forfeit the possibility to benefit somebody else [45–47]. Thus, we considered the *harm others* norm as a stronger modulator of behaviour than the *benefit to others* norm. Importantly, pay-off matrices were designed such that selecting the correct option (independent of social norms) would result in a maximization of pay-off for all players.

## 2. Material and methods

Participants (*N*_benefit other_ = 33 (male  =  15), *N*_harm other_ = 36 (male = 12), mean age = 27, range: 18–47) were recruited from the local participant pool and were paid on average 10 Euro, consisting of a show-up fee (5 Euro), a bonus for correct answers, and bonus and negative points collected by the other players (amounting to a variable remuneration from 4 to 7 Euro; see below). Twenty-six players participated for course credit, but nonetheless received payment for correct answers and bonus points. Participants reported no previous history of neurological or psychiatric illness.

### 2.1. Experimental task: dots under social influences task

**Figure 1.**
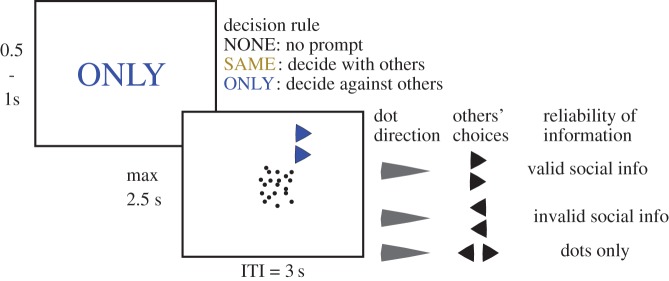
Dots under social influence task (DUST): Upper panel experimental set-up: Participants were tasked to judge the direction of motion in a random dot kinematogram. In addition to viewing the moving dots, players were also shown the decisions of two other players (social information, electronic supplementary material), that were allegedly taken from these individuals’ earlier responses. These decisions were depicted as arrows on top of the RDK. In two-third of the trials, players were prompted with two rules before each trial that would affect the remuneration of the other players, but not their own. Players were either prompted to decide with the other players (SAME), or they had to decide against the social information (ONLY). Players received no feedback on choice accuracy or additional points during the experiment.

In the experimental task, three or four participants per session decided whether dots in a random dot kinematogram task (RDK) were moving to the left or right (figure 1*a*). Players were all in the same room and conducted the experiment on their own computer without access to the other players' screens. In case a participant did not show up (2 sessions), we had a student assistant on stand-by that filled the empty slot. Experiments were split into two phases with 150 trials in phase one and 360 trials in phase two. The first phase was to familiarize participants with the set-up. In the second phase, we added social and normative information to this basic design.

In each trial during phase one, participants first saw the word ‘NONE’ in the centre of the screen for 0.5–1 s (figure 1*a*). The RDK was then displayed for up to 2.5 s until participants decided followed by an inter-trial interval of 3 s (1 s empty screen or feedback in case participant did not give an answer in time and a fixation cross for 2 s).

In this first phase, the difficulty was systematically varied by changing the dot coherence level to estimate at which level participants had approximately 70% correct choices. This was done for each participant individually. During stimulus presentation, two arrows were displayed on top of the random dots that pointed either in the same or in opposite directions. Arrows in phase one always pointed in opposite directions. A fixation cross in the middle of the screen appeared after the response. Participants received no feedback during the whole experiment whether their decision was correct.

In phase two, each correct decision regarding dot movement yielded one point (worth 1 cent). In this phase, we also added social information and norm prompts to the information from the RDK (figure 1 and table 1).

**Table 1.** Pay-off matrices norm conditions. Experiments only differed in their pay-off for other players in the *benefit to others* (BO) and *harm to others* (HO) condition. Numbers denote monetary consequences of a single decision in cent. This pay-off matrix was not revealed to players.

	SAME				ONLY			
social information	valid	valid	invalid	invalid	valid	valid	invalid	invalid
player decision	correct	wrong	correct	wrong	correct	wrong	correct	wrong
outcome self	1	0	1	0	1	0	1	0
outcome others *BO*	1	0	0	0	0	0	1	0
outcome others *HO*	0	−1	0	0	0	0	0	−1

### 2.2. Social information

We informed participants that we had collected their decisions during phase one and that randomly drawn decisions (with replacement) of two other participants would be displayed as arrows on top of the RDK. If four participants were present, we told them explicitly which two players' decisions they would see. Arrow stimuli were either incongruent (both pointing in different directions) or congruent (both pointing in the same direction). In the latter case, stimuli could be valid or invalid depending on whether they pointed in the direction of the random dots. In combination with the perceptual information, this resulted in three conditions regarding the presented information: dots only, valid social information and invalid social information (figure 1).

To keep social information constant across experimental groups, we presented fabricated social information. Nonetheless, we wanted to present realistic social information based on the average accuracy of players. We thus presented valid stimuli to all participants in 40% of the trials, dots only (arrows incongruent) and invalid information were each displayed in 30% of the trials. None of the participants raised suspicions about this manipulation. We, however, did not specifically ask participants whether they thought the information was fabricated.

### 2.3. Social norms

We implemented rules that participants had to follow to affect the remuneration of the other players. The same rules applied to both experiments. (Non)-compliance, however, affected the remuneration of the other players differently (see below). Rules applied to particular trials and were announced before each trial and additionally colour coded in the arrows displayed on the dot kinematogram. In addition to trials that had no particular rule, indicated by the word ‘NONE’ printed in grey, participants saw either the word ‘SAME’ printed in yellow or the word ‘ONLY’ printed in blue at trial onset. In the ‘SAME’ condition, players were prompted to decide in the same direction as the other two players (given of course that both arrows point congruently in the same direction). Contrarily, in the ‘ONLY’ condition, players were prompted to decide against the other two players (again under congruent arrows). Each condition had the same number of trials (*n* = 120).

Importantly, norms did not interfere with the optimality goal (to collect as many points as possible) because across all conditions the optimal strategy was to decide correctly on as many trials as possible. The pay-off matrix was not revealed to players prior to the experiment. Instructions made it clear, however, that the only way to score points was to select the correct option. We randomized norm trials with a restriction to a maximum of three identical conditions in succession. We tested two experimental groups under different social norm regimes to investigate how norm strength influenced perceptual processes.

### 2.4. Benefit others experiment

Here, players could collect bonus points (worth 1 cent) for the other players (but not for themselves). In the SAME condition, participants collected bonus points if they decided in the same direction as the social information, and all three players were correct in their estimate. In the ONLY condition, participants could collect bonus points if they decided against the social information and their estimate was correct. In the ‘NONE’ condition, participants could not collect bonus points. Beyond the bonus points, players again received points for themselves when deciding in the correct direction (table 1).

### 2.5. Harm others experiment

Instead of collecting bonus points, other players would get points (worth 1 cent) deducted if players incorrectly (with respect to dot motion) decided against the norm. Again, players received one point for answering correctly (table 1). In both experiments, participants had a mean accuracy of 70% when their decisions were based only on their individual percept (i.e. seeing only the RDK).

### 2.6. Statistical analysis

For sample size calculations, we made some simplifying assumptions. We were mainly interested in the added effects of norms over and beyond informational influences. We assumed that at least a 5% change in accuracy due to social norms would be meaningful with a standard deviation of 0.1 (medium effect size Cohen's *d* = 0.5). To detect such a difference with a dependent *t*-test, the minimum required sample size is 27 according to G*Power [48] at *α* = 0.05 and *β* = 0.2. We thus aimed at a number of participants between 30 and 40 per experiment.

We tested for differences in parameters of interest between experiments and conditions via linear mixed models in R using the lme4 [49] and lmerTest package [50] with Satterthwaite correction for degrees of freedom in *t*/*F*-tests. Contrasts were calculated via the multcomp package [51] using false discovery rate corrections for *p*-value adjustment [52].

### 2.7. Drift-diffusion models

To investigate how norms influence the perceptual decision-making process, we modelled accuracies and reaction times simultaneously via DDMs to test for differential effects of our manipulations on perceptual biases. The DDM is a mathematical model for two-AFC decisions. It assumes that evidence for a decision is continuously accumulated until one of two decision thresholds is reached where the rate of evidence accumulation is given by the drift rate. The evidence accumulation starts from a starting point that is located between the decision thresholds (see the electronic supplementary material, figure S3 for in-depth explanation).

We programmed and fit all models in R using Dynamic Models of Choice (DMC) software (http://www.tascl.org/dmc.html). DMC estimates model parameters using differential evolution Markov chain Monte Carlo sampling methods (DEMCMC [53]) and response time distribution functions provided by the rtdists package [54]. Given that the DMC package codes decision boundaries as being left or right, valid and invalid trials were re-coded to fit this categorization. Displayed parameters in the results correspond consequently to the mean of the two parameter estimates for left and right. For details on the MCMC sampling and chain convergence, see the electronic supplementary material.

To investigate the influence of perceptual information and social norms on perceptual decisions we compared six models. Throughout all models, we varied how two parameters in the drift-diffusion model were influenced by our experimental condition, the starting point bias and the drift rate. For an overview on the drift-diffusion model, see the electronic supplementary material, figure S3 and table S6 for the exact parametrization of each model with regard to starting point bias and drift rate. In the basic version (*Reduced*), we assumed that neither social nor normative influences affect the perceptual decision-making process. That is, this model consisted of the following free parameters: a single starting point bias for all conditions, threshold separation, mean drift rate, non-decision time and inter-trial-variability of drift rate. We then added two additional starting point parameters (for congruent left and right) for trials with congruent social information across all normative conditions (SP_reduced_). In an extension of this model (SP), we allowed this starting point bias to vary between conditions allowing for modulated biases in the norm conditions (adding two SP parameters for each condition). We also tested a model where we allowed the drift rate to vary by modelling a separate drift rate for incongruent and congruent social information trials (Drift_reduced_). We extended this model to allow for different drift rates between conditions (Drift). A final complex model combined the SP and Drift Model (SPDR). For a detailed table on the parameters included in each model, see the electronic supplementary material, table S6. We fit models to individual data, compared these via the Watanabe-Akaike Information Criterion (WAIC), and calculated the respective weights. We used these weights and averaged parameters across models for each individual [55,56] (for details see electronic supplementary material).

To check the validity of our DDM models, we also conducted analyses to recapture model parameters and conducted posterior predictive checks (see the electronic supplementary material). Briefly summarized, parameters could be recovered well and posterior predictive checks matched with observational data. See the electronic supplementary material for more detail.

In all experiments, we ensured that participants understood the rules properly by asking the group after phase one several questions regarding their correct understanding of how points and bonus points could be obtained via a short PowerPoint presentation. We openly announced the effect of bonus, respectively negative, points on their remuneration to each player after the experiment. During the experiment, there was no feedback on correct/incorrect decisions and bonus, respectively negative, points.

## 3. Results

**Figure 2.**
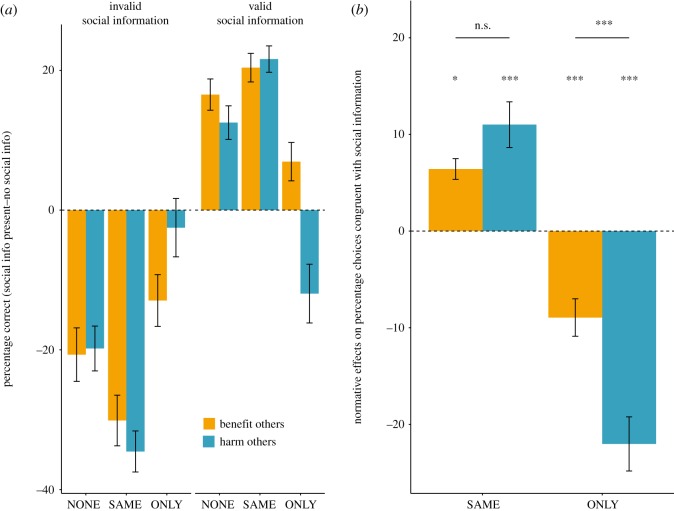
(*a*) Players used social information to guide their choices. Consequently, accuracy increased when valid social information was presented and decreased under invalid social information. Accuracy further increased when a norm prescribed a decision in line with social information (SAME). Contrarily, under a norm prompting decisions against social information the effect was attenuated (ONLY). (*b*) Conformity was modulated by social norms. Added social norms resulted in an increase (SAME) or decrease (ONLY) of choices in line with the social information (i.e. conformity) compared to a condition without social norm (NONE). This change was stronger in the *harm others* experiment when players were prompted to decide against the other players. Post hoc comparisons were conducted for each condition and experiment separately for deviation from zero and whether experiments differed within conditions. *p < 0.05; ***p < 0.001. In both panels, error bars denote standard error of the mean. For statistics in (*a*) and (*b*), see main text.

In phase one of the experiment, we adjusted coherence levels so that each player's performance would match 70% correct choices. In phase two, accuracy aggregated across dots-only trials (i.e. when players decided on perceptual information alone) approximately matched the targeted accuracies (Accuracy HO: Mean = 0.71, s.d. = 0.1; BO: Mean = 0.72, s.d. = 0.11). In a first step, we tested how norms affected players' accuracy (i.e. correct choices). Through the addition of social information, performance increased with valid and decreased with invalid social information compared to the dots-only condition, indicating that players in the two experiments integrated individual and social information (figure 2*a*, main effect *F*_1,335_ = 285.5, *p* < 0.001, see the electronic supplementary material, table S1 for details on the full linear mixed model). There was an interaction between the validity of social information and norms. In the SAME condition, the effects of social information were augmented, whereas in the ONLY condition this effect was attenuated compared to the NONE condition (figure 2*a*, *F*_2,335_ = 57.9, *p* < 0.001). Overall, modulation of choices through norms was stronger in the *harm others* experiment as indexed by a three-way interaction between experiment, condition, and social information (*F*_2,335_ = 7.9, *p* < 0.001, electronic supplementary material*,* tables S1 and S2 for reaction time analysis).

Beyond accuracy, we investigated the effects of norms on players’ propensity to decide in line with social information (i.e. conformity). We investigated the effect of norm prompt (SAME/ONLY) and experiment (BO/HO) on choices congruent with social information (the difference between social norms present and no norms condition; figure 2*b*). Participants chose more often in the direction of social information under normative influences that prompted participants to follow the social information and vice versa when prompted to decide against the social information (figure 2*b*). There was a marginally significant effect of a main effect of social norms (*F*_1,134_ = 3.77, *p* = 0.054) with significant interaction between social norms and experimental condition (HO, BO) (*F*_1,134_ = 16.4, *p* < 0.001; figure 2*b*; electronic supplementary material, table S2). That is, norms influenced choice behaviour in the direction of their respective prompts. This effect was stronger in the *harm others* than in the *benefit others* experiment in the ONLY condition (*z *= −4.233, *p* < 0.001).

**Figure 3.**
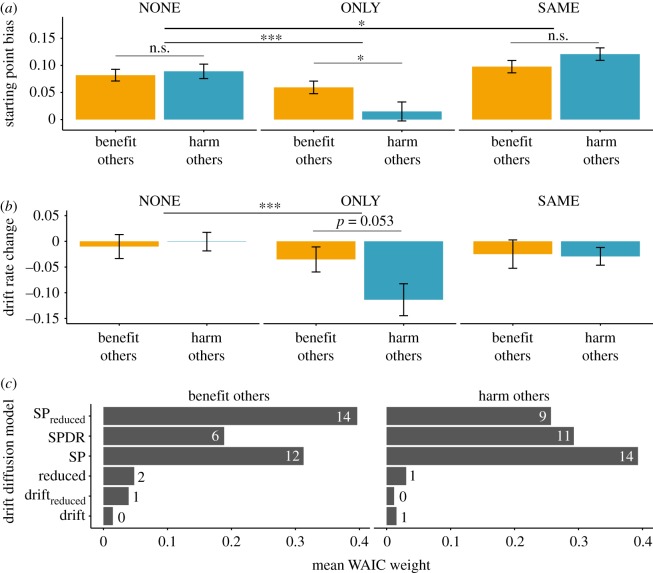
Parameters and model fit of DDMs of choice and reaction time data. (*a*) Starting point biases decreased when players were prompted to decide against the other players, particularly in the *harm others* experiment. Starting point values were calculated as means from estimates for left and right pointing social information, corrected for a potential bias in the incongruent condition and then weighted for each individual via their WAIC weight. (*b*) Drift rate decreased for the *harm others* particularly in the ONLY condition, but remained largely unchanged under all other conditions in both experiments. Depicted values are calculated as the difference between the drift rates in conditions with social information and the drift rate in the dots-only condition weighted for each model via the WAIC weights. (*c*) Mean WAIC weights (ranging from 0 to 1) for the six DDMs fitted to individual participants. Numbers indicate the number of participants for whom this model had the lowest WAIC. *p < 0.05; ***p < 0.001. Error bars denote standard error of the mean in (*a,b*).

In an additional step, we jointly analysed reaction time and choice data via a suite of DDMs. We investigated two parameters of interest, a starting point bias and the drift rate. We obtained parameter estimates for these two parameters for each individual and each condition via Bayesian model averaging (see Material and methods). We investigated differences via linear mixed models that investigated the effects of norm prompt (NONE/SAME/ONLY) and experiment (BO/HO). There was strong evidence that social information influenced decisions through a starting point bias (figure 3*c*). The magnitude of this starting point was similar in both experiments (*F*_1,69_ = 0.1, *p* = 0.75; figure 3*a*; electronic supplementary material, table S4). For a majority of players in the BO and HO experiments (*n* = 43; 60%, figure 3*c*), starting points were further adjusted under social norms. This resulted in a significant interaction between experiment and norm condition (*F*_2,138_ = 7.29, *p* < 0.001). Post hoc contrasts revealed that this was driven by the decrease of the starting point bias in the ONLY condition compared to the NONE condition (*z *= −5.2; *p* < 0.001) and an increase of the starting point bias in the SAME condition (*z* = 2.8, *p* < 0.05).

For the second parameter of interest, the drift rate, there was a significant interaction between norm condition and experiment (*F*_2,138_ = 4.56; *p* = 0.01; figure 3*b*; electronic supplementary material, table S5). A post hoc contrast revealed a significant decrease of the drift rate in ONLY condition compared to the NONE condition (*z *= −4.4, *p* < 0.001). In the ONLY condition, we found a decreased starting point bias in the HO experiment (*z *= −2.4, *p* < 0.05) and a decrease in the drift rate (*z* = −2.3; *p* = 0.053) compared with the BO experiment.

To investigate to which degree the observed effects depended on the reliability of the individual information, we conducted an additional experiment. In this experiment, the coherence of the RDK was increased but otherwise was identical with the HO condition. Here, participants showed an increased accuracy in the dots-only condition. Otherwise, informational influences and normative prompts showed similar effects as in the HO and BO experiment. We present the detailed analyses of this study in the electronic supplementary material. We also present an exploratory analysis of our data that investigates the effects separately for each gender in the electronic supplementary material, figure S1. This was inspired by a recent study on sex differences [57] in conformity.

## 4. Discussion

In this study, we investigated how social norms and perceptual and social information interact and how this affects perceptual decision processes. Choice behaviour and modelling analyses revealed that across two experiments participants integrated perceptual with social information. Participants chose frequently in the direction of the social information and consequently increased their correct choices under valid social information. Consequently, accuracy decreased under invalid social information. This bias was captured by a starting point bias in our DDMs and was further modulated by adding social norms that prompted players to decide with or against the social information. Whereas in the former case players increased their reliance on social information, players reduced their reliance on the social information in the latter case. Critically, the strength of this modulation was influenced by social norms. Particularly when non-compliance resulted in a negative outcome for the other players, participants used social information less when prompted to do so. This was underpinned by a stronger reduction of both response bias and drift rate than in conditions where compliance resulted in positive outcomes for players.

We show that, in line with previous work, the introduction of additional information, in this case social information, affects decisions via a starting point bias [40,41]. That is, responses were biased in the direction of the social information, directly influencing the amount of evidence (perceptual information) that is needed to elicit a response. This indicates a high reliance on the presented social information and reflects the coupling between reliability of social and individually acquired information. Even though participants in our experiments had no direct access to the reliability of social information, theoretical models support a reliance on social information that is linked to task difficulty [58–60]. That is, behaviours observed in others are meaningful and relevant to an optimality goal. Increasing relevance by highlighting that social models face similar conditions and constraints will result in an increase in compliance [18,61]. Contrarily, when this link cannot be established, social influences can even have an adverse effect [62].

We found no evidence, however, that drift rates changed through addition of social information alone (in the NONE condition), in contrast to earlier reports ([42,43] we refer to these studies as GM). A major difference between our study and the GM studies was the reliability of social information. Whereas in our study social information reliability matched approximately population accuracy, in the GM study the other players were chosen at chance level. Consequently, the authors in the GM studies describe the effect of an increased drift rate as a consequence of norms rather than information. The lack of a norms effect on a starting point bias in the GM studies is potentially due to the fact that social information did not facilitate uncertainty reduction. Consequently, there was no initial starting point bias as in our NONE condition. In the light of these findings, we suggest that social information elicits mainly a starting point bias that is afterwards modulated by norms.

Under social norms, starting point biases increased when norms prompted a decision in direction of the social information for most players. Contrarily, starting point biases decreased when norms mandated a decision against social information. Beyond starting point biases, norms affected perceptual processes, particularly in the ONLY condition where drift rates were decreased resulting in a slower information accumulation rate. The lack of such an effect in the SAME condition is potentially caused by a ceiling effect. The high response bias in this condition already causes decision accuracy to increase over 90% accuracy. This may have precluded the detection of a further increase in the drift rate.

In other studies on social influences, non-social control conditions were used to ascribe potential effects directly to social influences. In this experiment, a non-social control condition is not feasible as the effect of norms depends on the presence of other players. We emulated a non-social situation by adding a condition without consequences for the other players, but cannot exclude the possibility that even in the NONE condition, players chose in line with social information to comply with some norm unknown to the experimenter. Moreover, even though we use the term social information throughout the paper, we cannot rule out that players did not perceive the additional information as specifically social, but rather used it as abstract additional information. We also have no direct measure whether participants observed social information first during the simultaneous presentation of social and perceptual information, a necessary prerequisite for a starting point bias initialized at the start of the trial. Our results thus do not speak to the chronology of perceptual sampling processes during each trial.

Our results highlight the need to identify the effects of perceptual and social information alongside social norms in social influence studies as both result in choices that signify compliance, but with potentially different underlying neurocognitive mechanisms [63,64]. Studies investigating conformity potentially confound these two influence types as they predominantly manipulate information [24]. In a classic example, the Asch experiment, players decided in line with a unanimous majority in critical trials where confederates announced the obviously wrong answer. Here, the reliability of perceptual information was high and social information was also highly reliable (except for few critical trials). In the light of our findings, we suggest that in the Asch experiment, conformity in critical trials was elicited mainly by the available information. That is, social information (other player's choices) biased participants and this starting point bias potentially resulted in a long evidence accumulation time to elicit a correct response. For a possible effect of social norms, we speculate that a starting point bias was augmented by a shift from accurate to speeded responses [65]. As participants decided while others were watching, overly long reaction times may not contribute to a favourable self-image where hesitant answers deviate from the fast and ostensibly accurate answers of the confederates. In anonymous settings, such a reaction time comparison is not possible resulting in decreased conformity [22]. We thus suggest a careful reconsideration of findings on social influence to clarify whether social norms or available perceptual and social information guide behaviour.

Normative influences affected players' choices more strongly when non-compliance resulted in harm to others than if compliance led to benefits to others, particularly when participants were prompted to decide against social information. This adds to a growing literature that preventing harm to others is a strong modulator of human behaviour [45,66]. It also highlights an interaction between the reliability of information and social norms that result in a contextually guided integration of the two [31,67]. We suggest that norms are weighed against information akin to cue integration mechanisms where perceptual decision weights are adjusted by the respective relative reliabilities of each percept [68]. In the case of social norms, the utility of pursuing an optimality goal may be weighed against the utility of a compliance goal. In real-world scenarios, an investigation of both perceived information reliability and subjective norm strength in our view will yield important insights to predict behavioural outcomes of, for example, policy measures. Particularly in cases when behavioural change hinges on information conveyed by both group behaviours and institutional prescriptions (e.g. [69]).

## Supplementary Material

Additional Analyses and experiments

## Data accessibility

Experimental software, behavioural data and analysis scripts can be accessed under https://osf.io/kumrn.
