# Supplementary material for: Norm compliance affects perceptual decisions through modulation of a starting point bias
Source: R Soc Open Sci. 2018 Mar 28;5(3):171268. doi: 10.1098/rsos.171268 (PMC5882671; doi:10.1098/rsos.171268)
Supplement: Additional Analyses and experiments [file rsos171268supp1.docx]

**Supplementary information for**

**Normative influences modulate conformity through a static response bias in perceptual decision-making**

Ulf Toelch, Folco Panizza, Hauke R. Heekeren

**Table S1** Linear mixed effects model on change in percentage correct choices (compared to only dots condition). P-values calculated on df corrected by Satterthwaite correction. Refers to Figure 2A.  **(**HO: Harm others experiment, BO: Benefit others experiment)

|  | Numerator  DF | Denominator  DF | F-value | P-value |
| --- | --- | --- | --- | --- |
| experiment (HO/BO) | 1 | 67 | 1.74 | 0.1913 |
| CONDITION (NONE/SAME/ONLY) | 2 | 335 | 0.87 | 0.4191 |
| social information (Valid/Invalid) | 1 | 335 | 285.48 | <0.001 |
| experiment:CONDITION | 2 | 335 | 0.23 | 0.7940 |
| experiment:social | 1 | 335 | 6.66 | 0.0103 |
| condition:social | 2 | 335 | 57.90 | <0.001 |
| experiment:condition:social | 2 | 335 | 7.91 | <0.001 |

**Table S2** Linear mixed effects model on percentage choices in line with social information (Percentage choices with social information in norm conditions - Percentage choices with social information *without* norm condition). P-values calculated on df corrected by Satterthwaite correction. Refers to Figure 2B.  **(**HO: Harm others experiment, BO: Benefit others experiment)

|  | Numerator  DF | Denominator  DF | F-value | P-value |
| --- | --- | --- | --- | --- |
| experiment (HO/BO) | 1 | 134 | 3.77 | 0.0542 |
| CONDITION (SAME/ONLY) | 1 | 134 | 122.73 | <0.001 |
| experiment:CONDITION | 1 | 134 | 16.35 | <0.001 |

**Table S3** Linear mixed effects model on change in reaction times (compared to only dots condition). Includes all three (df different than above) experiments from Figure S2. P-values calculated on df corrected by Satterthwaite correction.

|  | Numerator  DF | Denominator  DF | F-value | P-value |
| --- | --- | --- | --- | --- |
| experiment (HO/BO/HO85) | 2 | 104 | 2.15 | 0.1218 |
| CONDITION (NONE/SAME/ONLY) | 2 | 520 | 31.73 | 0.0000 |
| social (Valid/Invalid) | 1 | 520 | 297.48 | 0.0000 |
| experiment:CONDITION | 4 | 520 | 2.00 | 0.0935 |
| experiment:social | 2 | 520 | 8.11 | 0.0003 |
| condition:social | 2 | 520 | 16.72 | 0.0000 |
| experiment:condition:social | 4 | 520 | 0.68 | 0.6053 |

**Table S4** Linear mixed effects model on Starting Point Bias (derived from Bayesian Model Averaging). P-values calculated on df corrected by Satterthwaite correction.

|  | Numerator  DF | Denominator  DF | F-value | P-value |
| --- | --- | --- | --- | --- |
| CONDITION (NONE/SAME/ONLY) | 2 | 138 | 31.62 | <0.001 |
| experiment (HO/BO) | 1 | 69 | 0.10 | 0.75 |
| Condition:experiment | 2 | 138 | 7.29 | <0.001 |

**Table S5** Linear mixed effects model on Drift Rate changes (Drift Rate in conditions with social information present – Drift Rate in dots only condition; derived from Bayesian Model Averaging). P-values calculated on df corrected by Satterthwaite correction.

|  | Numerator  DF | Denominator  DF | F-value | P-value |
| --- | --- | --- | --- | --- |
| CONDITION (NONE/SAME/ONLY) | 2 | 138 | 10.21 | <0.001 |
| experiment (HO/BO) | 1 | 69 | 0.72 | 0.4 |
| Condition:experiment | 2 | 138 | 4.56 | 0.01 |

**Analysis split for gender**


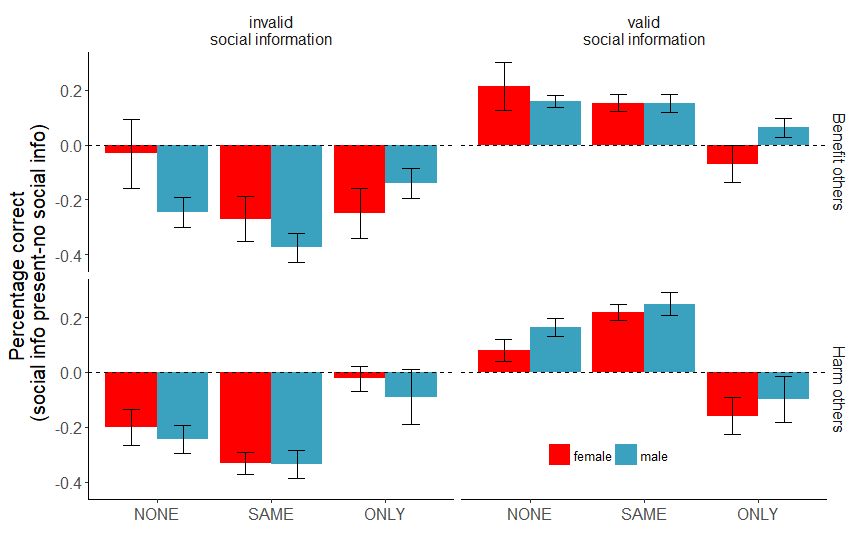


B

A


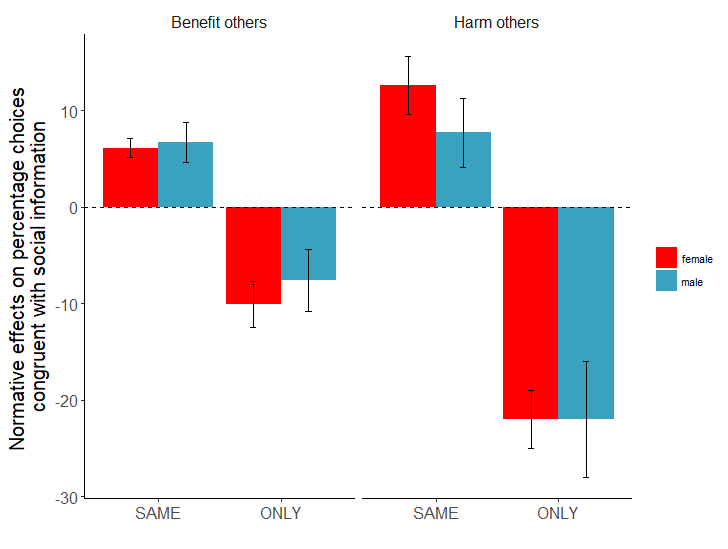


**Figure S1** **A.** Accuracy (percentage correct choice) displayed separately for female and male participants. **B.** Choices in line with social information displayed separately for male and female participants. Compare to Figure 2.


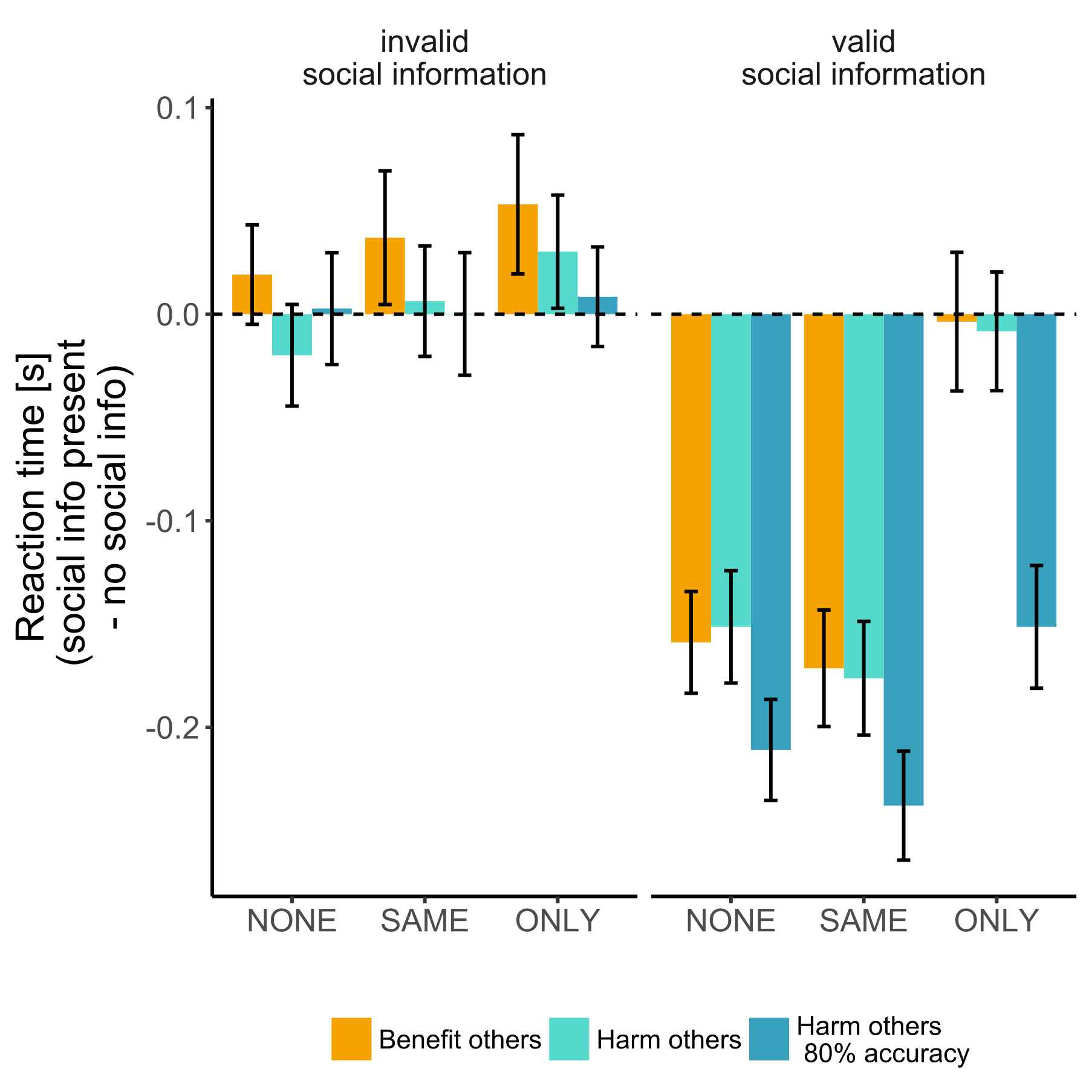


**Figure S2** Reaction time differences between conditions with social information and dots only condition across all three experiments.

**Fitting Drift Diffusion models**


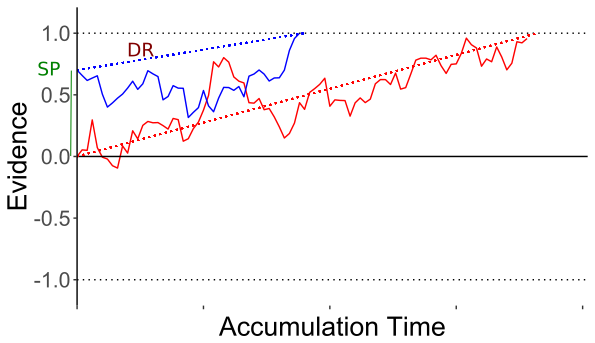


**Figure S3** Drift Diffusion Model (DDM) for a perceptual decision. Evidence is accumulated over time for one option over the other with a random component modelling sensory noise. Depicted here are the two main parameters of interest for our DDMs that varied between models. SP (green) denotes a starting point bias that in our case indexes the strength of a bias in the direction that the available social information is indicating. The drift rate (DR) relates to the amount of evidence that is accumulated per time unit (the slope of the coloured dotted lines). A response is elicited once enough evidence is accumulated to hit one of the thresholds. In the blue case, less evidence is needed (at a similar drift rate) to elicit a response due to the presence of a starting point bias.

**Table S6** Parametrisation of Drift Diffusion Models. Models differed in respect to their starting point biases (SP) and drift rates (DR). Numbers reflect different parameters for each condition. Parameters depicted in Figure S5 remained the same across all conditions.

|  |  | Social info present | | |  | Social info present | | |
| --- | --- | --- | --- | --- | --- | --- | --- | --- |
|  | SP | | | | DR | | | |
| Model | Dots  Only | NONE | SAME | ONLY | Dots only | NONE | SAME | ONLY |
| Reduced | SP1 | SP1 | SP1 | SP1 | DR1 | DR1 | DR1 | DR1 |
| SP red. | SP1 | SP2 | SP2 | SP2 | DR1 | DR1 | DR1 | DR1 |
| SP | SP1 | SP2 | SP3 | SP4 | DR1 | DR1 | DR1 | DR1 |
| Drift Re | SP1 | SP1 | SP1 | SP1 | DR1 | DR2 | DR2 | DR2 |
| Drift | SP1 | SP1 | SP1 | SP1 | DR1 | DR2 | DR3 | DR4 |
| SPDR | SP1 | SP2 | SP3 | SP4 | DR1 | DR2 | DR3 | DR4 |

*Prior selection*

Prior distributions for the decision boundary *a*, drift rate *v*, and drift rate standard deviation *sv* were set to be truncated normal, centred in the middle of their theoretical range (*a*=1, *v*=0, *sv*=1) and with a standard deviation of 1, 2 and 2 respectively. Starting point *z* and decision time *t0* priors were beta distributed, with α=2 and β=2 for *z*, and a uniform distribution for *t0* (α=1, β=1). Due to a failure in the recovery of the starting point standard deviation *sz*, we fixed this parameter at a constant value of 0.11.

*Number of iterations and convergence*

Based on multivariate Gelman hat estimates ($\hat{R}$; Gelman and Rubin, 1992), we determined the number of DE-MCMC iterations to be 1600, with a burn-in period of 400 iterations with chain migration set at 5%. Burn-in was flexibly extended in the occurrence of significant convergence failures. With these settings, all model estimates reached a convergence approximation of 1.1 (Gelman, Carlin, Stern & Rubin, 2004), with the exception of the SPDR model, whose estimates ranged up to 1.2. This notwithstanding, we left the number of iterations unaltered, given the good recovery performance (see Parameter recovery).

*Model averaging*

We fitted six models to reaction time and choice data. Our parameters of interest were the drift rate and the starting point bias. For each model, we obtained these parameters for each condition. We averaged over these parameters. That is, each parameter *θ_i_* present in our model space with *R* models was weighted via their WAIC weights (Eq. S1). In some models, parameters were not estimated for each normative condition, but were fitted to the pooled responses. In these cases, we used this parameter for all other conditions as well. For example, the *Reduced* model had no separate starting point for each condition. When calculating the average parameters, the starting point bias was the same across all conditions (see Table S4). For estimating the drift rate in the NONE condition, the parameters from the respective column in Table S4 are weighted by their WAIC weight and the summed up and divided by the number of models (in our case six).


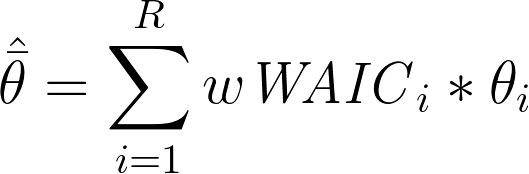
 Eq. S1

*Parameter recovery*

For each drift diffusion model and for both levels of difficulty, we tested the ability of the DMC software to correctly recover parameter values. To this end, a set of reaction times and accuracy ratings (N=49 for each model version) was simulated from predetermined parameter values and refit by the DDM algorithm. Values recovered from these choice data were correlated to the original parameter values. Supplementary table 1 presents the correlation estimates: recovered values of *a*, *v*, and *z* parameters strongly correlated with the original values in all model versions and for both difficulty levels (all *R*s>0.98, df = 28, all *p*s<0.001), and the *sv* parameter recovered reasonably well with all *R*s between 0.67 and 0.78 (all *p*s<0.01). In addition, we calculated the difference between original and recovered values to test the degree of biasedness of the recovery: this measure confirmed that there was no systematic bias for any of the models (all *p*s>0.01, all *t*s(29)>2.58).

**Table S7**. Correlation measures between simulated and recovered data.

|  | ***A*** | ***v*** | ***Z*** | ***sv*** | ***t0*** |
| --- | --- | --- | --- | --- | --- |
| RE_70_ | ≈1 | ≈1 | ≈1 | 0.67 | ≈1 |
| RE_80_ | ≈1 | ≈1 | ≈1 | 0.74 | ≈1 |
| SPr_70_ | ≈1 | ≈1 | ≈1 | 0.73 | ≈1 |
| SPr_80_ | ≈1 | ≈1 | ≈1 | 0.74 | ≈1 |
| DRr_70_ | ≈1 | ≈1 | ≈1 | 0.75 | ≈1 |
| DRr_80_ | ≈1 | ≈1 | ≈1 | 0.77 | ≈1 |
| SP_70_ | ≈1 | ≈1 | 0.98 | 0.76 | ≈1 |
| SP_80_ | ≈1 | ≈1 | 0.98 | 0.73 | ≈1 |
| DR_70_ | ≈1 | 0.98 | ≈1 | 0.69 | ≈1 |
| DR_80_ | ≈1 | 0.99 | ≈1 | 0.78 | ≈1 |
| SPDR_70_ | ≈1 | 0.98 | 0.98 | 0.78 | ≈1 |
| SPDR_80_ | ≈1 | 0.99 | 0.98 | 0.76 | ≈1 |

*Posterior predictive check*

We employed a posterior predictive check (Gelman et al., 2004) in order to assess the accuracy of the models in retrieving empirical data. Through this procedure, reaction times and responses were generated from the parameter estimates and plotted against the original values averaged across participants. Model-generated data matched original reaction times well.

**Figure S4.** Posterior predictive check for the SP model


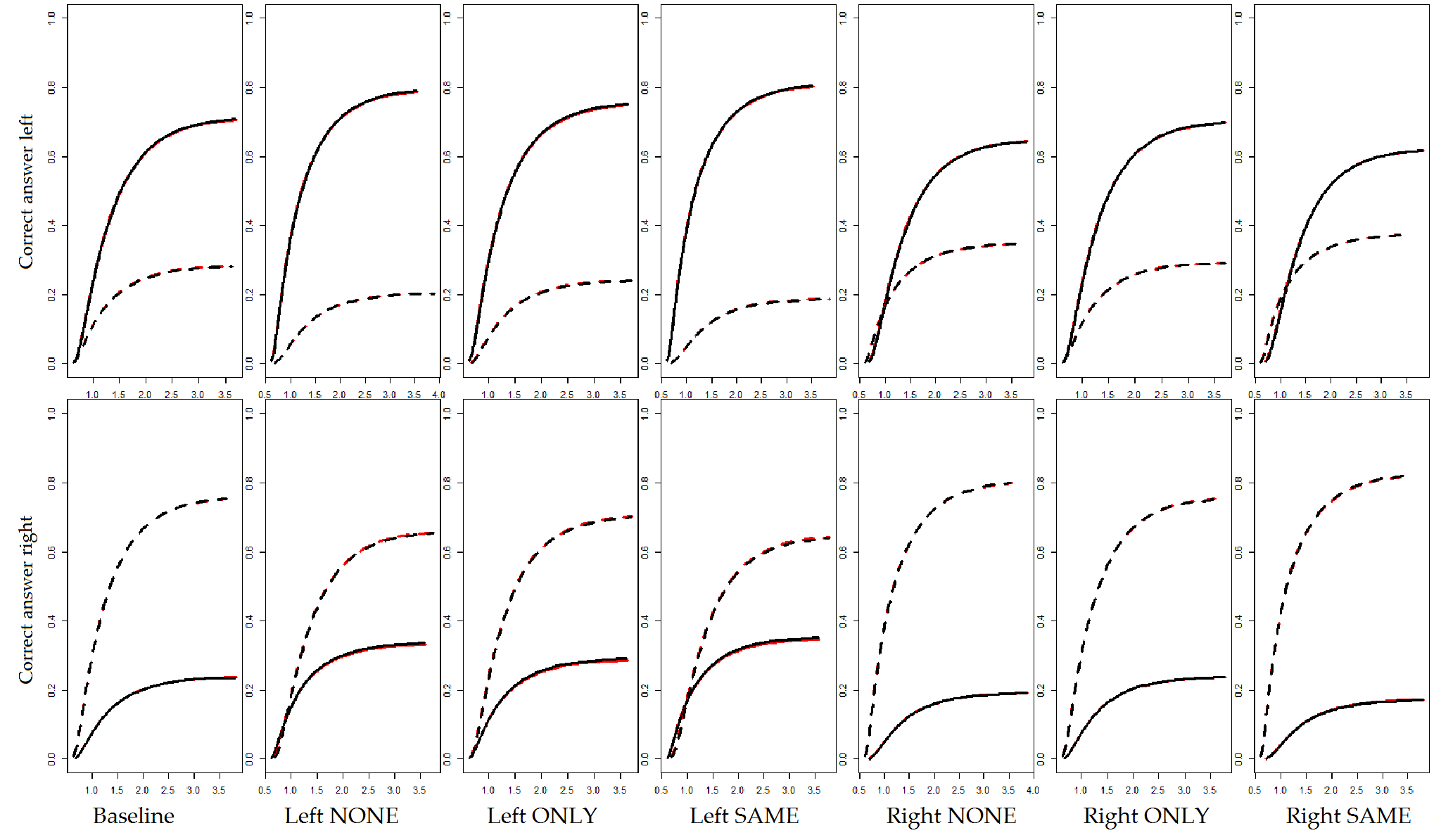


Post-predictive generated (red lines) and original (black lines) data distributions are summarised using percentiles and smoothed in defective cumulative distributions (Ratcliff & Tuerlinckx, 2002). Each plot represents data in one condition (e.g. left cue, NONE), and with either left or right as the correct answer. Time (*s*) is represented on the x axis, cumulative density on the y axis. Continuous lines indicate left answers, dashed lines indicate right answers.


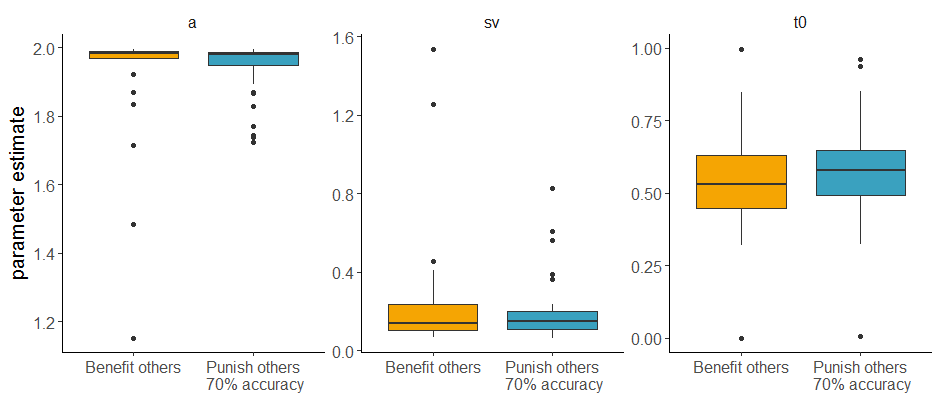


**Figure S5** Additional parameter estimates derived from drift diffusion models. a=Threshold separation, sv=standard deviation of drift rate, t0=non decision time. There were no differences in these parameters between experiments.

**Additional experiment: Increasing accuracy in the RDM task**

In many experiments on norm compliance, uncertainty in the perceptual task is low. To test whether the effects identified in the previous experiments hold for higher accuracy levels (i.e. low perceptual uncertainty), we manipulated the strength of individual information by increasing dot coherence. Players’ (n_80_=38) difficulty was adjusted to 80% accuracy (instead of 70%). Again, we adjusted the reliability of the presented social information to the perceptual accuracy level, as it is more likely to see valid social information under low perceptual uncertainty. We presented valid stimuli to all participants in 62% of the trials, 23% invalid and 15% incongruent trials. In phase two, players played 396 trials in this experiment instead of the 360 trials in the other experiments. The number of trials was slightly increased to have a sufficient number of trials with invalid information. In all other respects this experiment was identical to the *harm to others* experiment.

*Results*

When increasing accuracy in an additional experiment to approximately 80% correct choices in the dots only condition (Mean= 0.78, SD = 0.12) results regarding the effect of normative influences were robust albeit slightly alleviated. With regard to the percentage correct choice compared to the dots only condition, we again found a significant interaction between social information (valid/invalid) and normative condition (Figure S4A, *F*(2,222)=13.1, *p*<0.001). This resulted in increased choice congruent with social information in the SAME condition and a decrease of this proportion in the ONLY condition when compared to the NONE condition (Figure S4B, *F*(1,74)=23.5, *p*<0.001). Post hoc contrast indicated that both changes were significantly different from zero although the SAME condition was only close to our criterion (SAME: *t*(74)=1.98, *p*=0.052; ONLY: *t*(74)=-4.9, *p*<0.001).


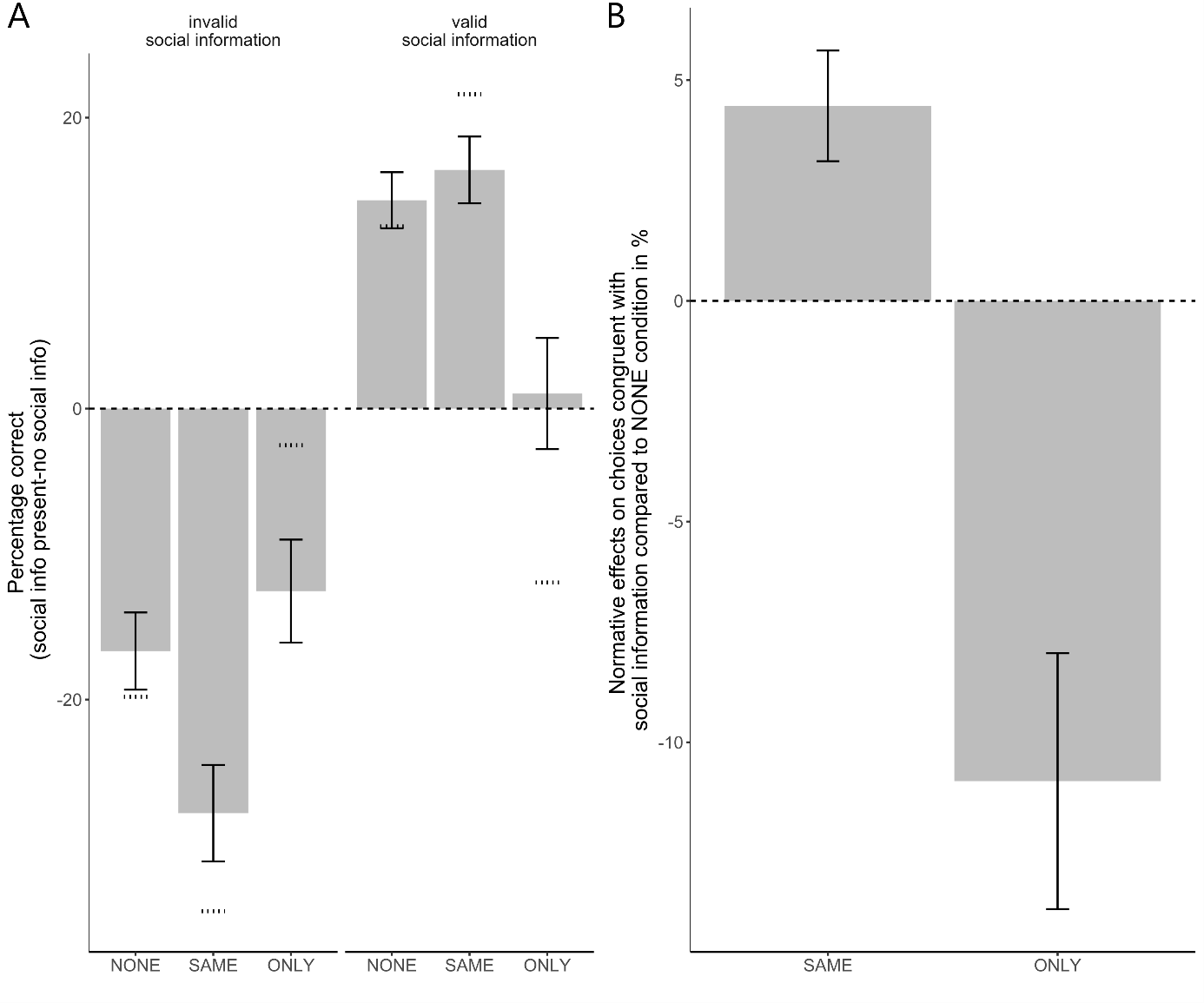


**Figure S6** **A.** Effects observed in the two main experiments were preserved in a control experiment (N=38) where perceptual uncertainty was reduced. Again, accuracy was increased under valid and decreased under invalid social information indicating use of social information. A comparison with the *harm others* experiment under high perceptual uncertainty (dotted lines equivalent to *harm others* in Figure 2A, main text) showed higher reliance on informational than on normative effects. **B.** Social norms increased choices congruent with social information in the SAME condition and decreased these choices in the ONLY condition. Error bars denote standard error of the mean in both panels. For statistics see above.

SUPPLEMENTARY REFERENCES

Gelman, A., & Rubin, D. B. (1992). Inference from iterative simulation using multiple sequences. *Stat. Sci.*, 457-472.

Gelman, A., Carlin, J. B., Stern, H. S., & Rubin, D. B. (2004). Posterior simulation. *Bayesian Data Analysis. Boca Raton, FL: Chapman and Hall/CRC*, 283-310.

Ratcliff, R., & Tuerlinckx, F. (2002). Estimating parameters of the diffusion model: approaches to dealing with contaminant reaction times and parameter variability. *Psychon. Bull. Rev.* 9, 438–481.
